# How to Sign a Prescription: The Latest Insurance Act Problem

**Published:** 1915-05-22

**Authors:** 


### HOW TO SIGN A PRESCRIPTION.

### The Latest Insurance Act Problem.

Among the problems of conduct raised by the Insurance Act one of the latest relates to the proper method of signing a prescription. The question has lately agitated the authorities in Kent, but even in this limited sphere it does not appear to have reached a settlement. The Medical Benefit County Sub-Committee advised that doctors in their own interests should sign their prescriptions, and to this there was, in the debate, little or no objection. But then arose the question, What is a signature? Must the full name be written, or will initials suffice? If something more than initials is required, will the ordinary signature be satisfactory, or must the complete baptismal endowment be displayed? Again, with what materials must the writing be effected? Is pen and ink essential?

All these questions, it seems, arise when, entangled in the meshes of the law, the medical practitioner indulges in so commonplace an event as writing a prescription. One of the members of the Kent Insurance Committee advises that if a prescription signed with initials only is presented to a pharmacist it should be sent back for the doctor's "signature"; and another contends that initials would not be accepted in a court of law. This latter statement is perhaps a little too confident. Courts of law not infrequently accept evidence as to trade or professional custom, and in certain directions they attach great weight to practices which can claim this definition. Further, there is probably some considerable legal value in the Apothecaries Act of 1815, one of the clauses in which deals specifically with the question now in debate. It is there enacted that any apothecary who shall refuse to compound any medicines "directed by any prescription order or receipt *signed with the initials* in his own handwriting" of any physician licensed to practise physic by the president and commonalty of the Faculty of Physic in London, or by either of the two Universities of Oxford or Cambridge, shall be liable to be fined, and on repetition of the offence may lose his licence as an apothecary. It may be difficult nowadays to say exactly what is an "apothecary," and we will not pretend to say that a pharmacist or member of the Pharmaceutical Society certainly comes under this designation. But in view of professional custom and of the terms of the Apothecaries Act it would seem quite possible that a licentiate of the College of Physicians of London, or a medical graduate of Oxford or Cambridge, could make a good legal fight for the directing power of his initials.

Another interest in reference to the signing of the prescription is provided by what may be called classical tradition. In days more stately and formal than the present there were precise rules on the subject. The custom was for a physician to sign with his initials except when he was called upon to prescribe for a member of the Royal Family, where etiquette demanded that he should write his name in full. We can remember an encouraging lecturer who used to advise all his pupils to be prepared for this extended responsibility.

On the other hand, the surgeon, when he ventured to undertake the risk of prescribing, was understood to write his surname in full, but to employ only the initials of his baptismal name. These rules or habits applied to what was called the sign-manual of the prescription, for in classical times the *signatura* of the prescription referred, not to the final flourishes, but to the part of the document which defined the directions to the patient. The formal model of the prescription included first the *invocation*, now represented by the symbol ℞, and usually translated as *Recipe*, though, at least on one occasion, expressed by a hesitating student in something like its original meaning, when, contemplating the blank paper before him, he exclaimed, "Oh! Jupiter, help us!" Following the invocation came the materials to be employed, *materiæ designatio*, and these were to be set out in due order as *basis* or principal medicine; *adjuvans*, helping or promoting the action of the basis; *corrigens*, correcting any undesirable quality of the other medicines; and finally the *vehiculum*, or medium in which the various remedies were presented in a form suitable for administration. The third part of the prescription was the *subscriptio*, or directions to the compounder, and following this was the *signatura*, or directions to the patient. Thereafter came the patient's name, and the date of the event, and the solemn ceremony was closed with the *sign-manual*, as already described.

Nowadays we have largely escaped from the formalities of these severe exercises, though perhaps not wholly without loss. Is it not, for example, possible that there is too ready a disposition to fall back upon a vague "To be taken as directed," rather than to write out in detail the exact course recommended? There are, no doubt, occasions when delicacy may forbid precise details written in a prescription open to the contemplation of curious eyes. Thus we should sympathise with the embarrassment of a pharmacist who found himself confronted with the necessity of expressing in prosaic ink an English equivalent for *urgente borborygmo*. But because, at times, directions must be left with the patient or a nurse, this is not to say that it is wise to write a general phrase and to define it purely by word of mouth. In other words, we have something to learn from the precise and accurate prescribing habits of our forefathers. To come back to our original point, in the practical world the physician had better authorise his prescription by means of his usual signature. When a prescription was almost invariably dispensed in a narrow geographical area, initials, even though they assumed fantastic forms, had a meaning which was easily identified. But this is no longer true, and the dispenser may reasonably expect the authority for a prescription to be presented in a form capable of secure verification.

